# Healthcare Professionals’ Perceptions and Acceptance of Telemonitoring During Pregnancy and Early Labor: A Single-Center Survey

**DOI:** 10.3390/ijerph22111753

**Published:** 2025-11-19

**Authors:** Julia Jockusch, Sophie Schneider, Andrea Hochuli, Marianne Simone Joerger-Messerli, Daniel Surbek, Anda-Petronela Radan

**Affiliations:** 1 Department of Obstetrics and Gynecology, University Hospital Bern, University of Bern, 3010 Bern, Switzerland; julia.jockusch@mhb-fontane.de (J.J.); sophie.schneider@insel.ch (S.S.); andrea.hochuli@insel.ch (A.H.); daniel.surbek@insel.ch (D.S.); anda-petronela.radan@insel.ch (A.-P.R.); 2 Department of Prosthetic Dentistry and Senior Dentistry, Brandenburg Medical School (Theodor Fontane), 14776 Brandenburg an der Havel, Germany; 3 Department for BioMedical Research (DBMR), University of Bern, 3008 Bern, Switzerland

**Keywords:** telemedicine, telemonitoring, perception, survey, midwife, obstetrics, neonatology, prenatal nurse care, wearables, devices

## Abstract

The use of health monitoring software applications (apps) and devices is gaining popularity in obstetrics. The attitude and acceptance of different healthcare professionals regarding telemonitoring during pregnancy and the early phase of labor have not been sufficiently investigated. This study aims to assess healthcare professionals’ views on telemonitoring during pregnancy and childbirth, as well as data processing in the telemonitoring process. The study is part of an international project called `Newlife`, funded by the European Council and nationally funded by the Swiss State Secretariat for Education, Research and Innovation and Innosuisse. Eleven physicians from the fields of obstetrics and neonatology and five prenatal care nurses and five midwives were interviewed. First, participants were asked to fill out a written questionnaire with open and closed-ended answers, containing questions with a 5-point Likert scale. In a second step, a personal oral interview was conducted with all respondents. The study had an exploratory, qualitative focus. Questionnaire responses were summarized using descriptive statistics, while interview recordings were transcribed verbatim and systematically coded to identify recurring themes. Of the respondents (*n* = 20), five (25.0%) reported previous experience with telemonitoring in their professional practice, and all of them considered it useful. Regarding attitudes and acceptance, 57.1% (*n* = 12) of respondents would welcome telemonitoring during pregnancy and 52.4% (*n* = 11) during the early phase of labor, while 33.3% expressed no clear opinion. Rejection of telemonitoring was indicated by 9.6% (*n* = 2) during pregnancy, and 19.0% (*n* = 4) during early labor. In terms of perceived benefits, respondents highlighted early detection of problems (*n* = 13, 61.9%), improved prenatal care (*n* = 11, 52.4%), and better opportunities for data analysis and research (*n* = 12, 47.1%). Perceived risks included technical challenges and susceptibility to errors (*n* = 14, 66.7%), the lack of human contact and personal support (*n* = 14, 66.7%), and potentially inaccurate measurements (*n* = 12, 57.1%). This study offers insights into healthcare professionals’ attitudes and acceptance of telemonitoring in healthcare during pregnancy and the early stages of labor. There is a generally positive outlook but concerns and preferences exist. Addressing these considerations is essential for developing effective and user-friendly telemonitoring systems that benefit both healthcare professionals and pregnant women.

## 1. Introduction

The use of health monitoring software applications (apps) and telemonitoring devices, or so-called wearables, has rapidly gained popularity in the past decade [1,2,3]. Wearables are portable devices that often contain sensors and can collect data on various health parameters. Many companies offer personalized health trackers and market them as convenient lifestyle products for daily use. Accordingly, monitoring tools for nutrition, menstrual cycle, and pregnancy diaries are widely available [4,5].

Concurrently, increasing rates of both urgent and non-urgent emergency visits have been observed in obstetrics and across other areas of medicine. [6]. Common reasons for emergency consultations include concerns and anxiety about the unborn child. In such cases, it is often difficult for patients to assess whether a particular condition is pathological or physiological, which can lead to a higher number of consultations. Studies show that pregnant women are often discharged from the emergency department despite feeling a high level of urgency because no pathologic findings were noted [7,8]. Data from prolonged home monitoring could potentially have diagnostic value in such situations. It is also conceivable that these additional data could allow healthcare professionals to remotely reassure patients and their partners in the future.

In 2018, a total of 38% of the hospitals in the Netherlands offered telemedical services such as home monitoring, remote monitoring, or both as an alternative to hospital admission for women with pregnancy complications [9]. Telemedicine refers to the provision of medical services and information via telecommunications and information technologies [9]. Since then, two major clinical trials in the Netherlands—SAFE@home [10] and the HoTeL Trial [11]—have demonstrated safety and effectiveness of telemonitoring in pregnancy care. Furthermore, the PregnaDigit EU initiative has been launched to implement remote digital pregnancy care for high-risk pregnancies in the Netherlands, Spain, and Sweden [12]. Further research highlights the use of wearable devices for remote pregnancy monitoring, emphasizing the growing demand for such technologies [13,14,15]. The implementation of structured home surveillance during pregnancy, guided by established protocols, has the potential to enhance the accuracy of maternal and fetal monitoring, provide reassurance to expectant mothers, and improve the early identification of high-risk cases—representing a meaningful advancement in obstetric care [16].

Development of such technologies is only one part of the equation, as acceptance and medical purpose must be addressed, including creating practice guidelines where telemedicine is included to some extent as part of standard care. However, these aspects are still fairly under development, as only few studies address the attitudes and perceptions of pregnant women and clinicians towards wearable devices for home monitoring in pregnancy care [17,18,19,20].

Obstetric health professionals’ attitudes toward telemedicine remain mixed, largely influenced by their individual attentiveness and the perceived convenience of the technology [20,21]. Yet, since the COVID-19 pandemic, which has accelerated the introduction of telemedicine, the attitude of health professionals towards telemedicine has shifted positively and is expected to further increase in popularity [22]. Studies show that both patient acceptance and clinician satisfaction were significantly higher in clinics already using telemedicine. Prior use was also positively linked to expected long-term adoption [17]. Most patients considered telemedicine appointments as equal in quality to in-person visits [17].

While the Swiss healthcare system is recognized for its excellence, it remains challenged by comparatively slow digital transformation, most notably in the field of perinatal care. A recent survey of 1160 Swiss women found that those living in rural areas had a more negative attitude toward digital health in perinatal care compared to women in urban settings [23]. However, the attitudes and perceptions of Swiss healthcare professionals toward telemedicine in pregnancy and early labor remain very limited.

Thus, the aim of our survey study is, first, to evaluate the attitude and acceptance of different healthcare professionals regarding the telemonitoring of pregnancy and the early labor in a tertiary care hospital in Bern, Switzerland. Secondly, the study aims to elicit professional opinion on telemonitoring in pregnant women with and without risk factors and on data processing in the process of telemonitoring.

## 2. Materials and Methods

We employed a qualitative descriptive design to explore healthcare professionals’ preferences, needs, and expectations regarding remote monitoring during pregnancy and early labor.

The survey was conducted from August to October 2023 among different healthcare professionals on the topic of telemonitoring in pregnancy and the early phase of labor at the University Hospital Bern, Switzerland. The study is part of an international project called `Newlife`, funded by the European Council and nationally funded by the Swiss State Secretariat for Education, Research and Innovation and Innosuisse (www.newlife-kdt.eu, accessed on 24 June 2025).

For research purposes, healthcare professionals from different professional groups were interviewed: eleven physicians from the fields of obstetrics and neonatology, five prenatal care nurses and five midwives. Due to the exploratory and qualitative nature of the study, a relatively small sample was recruited using purposive sampling to include key professional groups involved in pregnancy monitoring and care. Participants were recruited through purposive sampling to ensure adequate representation of key professional groups directly involved in pregnancy monitoring and care.

The initial target number of participants was five experts per professional group. As one participant in the group of the obstetrics physicians did not fully complete the questionnaire, an additional person was recruited for this professional group. All the assessed information was used in the analysis.

The study consisted of two parts: a written questionnaire and an oral interview. The questions were developed through a review of relevant literature, adaptation of previously validated and published questionnaires [24], and expert discussions within the research team to ensure content validity and alignment with the study objectives.

First, participants were asked to fill out a written questionnaire using the survey platform Survey Monkey [25] (Supplementary File S1). The questionnaire was structured in nine sections, including (A) Background Information on the Specialist, (B) Options, Preferences, and Requirements related to Remote Monitoring, (C) Assessment of Parameters in Pregnant Women with Risk Factors, (D) Assessment of Parameters in Pregnant Women without Risk Factors, (E) Data Management, (F) Data Processing, (G) Requirements for the Remote Monitoring Device, (H) General Questions about Remote Monitoring Devices, and I) Attitude towards Remote Monitoring. The questionnaire primarily consisted of closed-ended questions, including “Yes/No/No opinion” items, checkbox questions that allowed multiple selections, and Likert scale items designed to assess levels of agreement or frequency (e.g., “fully agree, agree, partly/partially agree, do not fully agree, do not agree at all” or “very important, important, partly/partly, rather unimportant, unimportant”). The time required for the written assessment per respondent was 45–60 min.

In the second step, a personal oral interview was conducted with all respondents based on the written questionnaires’ topics (Supplementary File S2). The aim of the interview was to explore the preferences, requirements, needs, and goals or expectations of healthcare professionals regarding remote monitoring systems during pregnancy and early labor. The interview was structured in three parts: (1) Understanding current practices and workflows during pregnancy and early labor; (2) Understanding opportunities for improvement; (3) Understanding user requirements and expectations for remote monitoring. All questions were open-ended to encourage in-depth responses. Interviews were recorded using a small recording device for subsequent transcription and analysis. The time required for the oral assessment per respondent was 45–60 min. The interviews were conducted with two interviewers and subsequently transcribed based on the reflexive thematic analysis of Braun and Clarke [26].

In both the written and oral interviews, questions were asked about the content of (a) healthcare professionals’ attitudes and acceptance of telemonitoring of pregnancy and the early phase of childbirth, (b) telemonitoring in pregnant women with and without risk factors, and (c) data processing in the process of telemonitoring.

### 2.1. Statistical Considerations

The collected data were analyzed using the statistical program SPSS version 27.0, IBM, Chicago, IL, USA [27]. Descriptive statistics (frequencies, percentages, mean and standard deviation (SD), median, and range) were applied to provide a summary of participants’ responses obtained from the questionnaire data. To examine potential differences between healthcare professionals’ perceptions in relation to high-risk and low-risk pregnancies, analysis of variance (ANOVA) test was conducted. Although the study had an exploratory and qualitative focus, the analysis helped identify statistically significant differences in attitudes and preferences between groups. The significance level was set at *p* < 0.05.

A qualitative content analysis method was used to analyze the interview data. The interview recordings were transcribed verbatim, and the resulting transcripts were systematically coded to identify recurring themes and patterns reflecting healthcare professionals’ perceptions, needs, and expectations regarding remote monitoring. All multiple responses were grouped into categories to facilitate analysis.

### 2.2. Ethical Considerations

The need for ethics approval for the study was waived by the cantonal ethics committee of Bern, Switzerland, as the study qualifies as an opinion survey involving healthcare professionals, with no collection of health-related data nor intervention. The study does not produce generalizable findings and is thus not subject to the provisions of the Human Research Act (Art. 2 and 3 HRA).

Participants provided oral informed consent, including explicit permission for audio recording of the interviews. All audio recordings and data were securely stored in password-protected files accessible only to the research team to ensure confidentiality and data privacy.

## 3. Results

The data are organized into two main sections—first the written questionnaire, and then the oral interviews—corresponding to the sequence in which they were administered.

The objective of the questionnaire was to address background information, preferences and requirements related to remote monitoring, assessment of clinical parameters in high-risk and low-risk pregnancies, data management and processing, device requirements, general questions about remote monitoring, and attitudes toward its use (Supplementary File S1). The questionnaire primarily included closed-ended questions, which directly allowed for quantitative analysis of healthcare professionals’ perspectives.

The objective of the oral interviews was to gain a deeper understanding of healthcare professionals’ preferences, requirements, needs, and expectations regarding remote monitoring systems during pregnancy and early labor (Supplementary File S2). The interviews covered both high-risk and low-risk pregnancies, as well as various pregnancy-related pathophysiological conditions, using open-ended questions to encourage detailed responses.

A total of 21 healthcare professionals (age: Mean ± SD: 44.1 years ± 9.5 years; Median (range): 44 years (32–61 years)) participated in the written survey. All of them were also interviewed orally. All participants worked in a university setting and had a mean work experience of 19.8 years ± 10.6 years (Median (range): 17 years (1.5–37 years)). The healthcare professionals (*n* = 21) consisted of obstetric (*n* = 6) and neonatal (*n* = 5) physicians, prenatal nurses (*n* = 5), and midwives (*n* = 5).

### 3.1. Written Survey

#### 3.1.1. Attitude and Acceptance of Healthcare Professionals Towards Telemonitoring During Pregnancy and the Early Phase of Labour

Of 20 respondents, 25.0% indicated that they had already used telemonitoring in some form in their previous professional life. All of them found this application useful.

Telemonitoring would be welcomed by 57.1% of 21 respondents in pregnancy and 52.4% in the early phase of birth, i.e., at the beginning of labor, premature rupture of membranes, or the latent phase. However, the decision to use remote telemonitoring should be guided by medical judgment, as one participant emphasized: “In my opinion, the application should be indicated by healthcare professionals. The motivation for application should not come from the pregnant woman herself.” One-third of the respondents (in pregnancy: 33.3%; in the early phase of labor: 28.6%) have no opinion or cannot assess this, while 9.6% reject it during pregnancy, and 19.0% in the early phase of labor, respectively.

**Figure 1:**
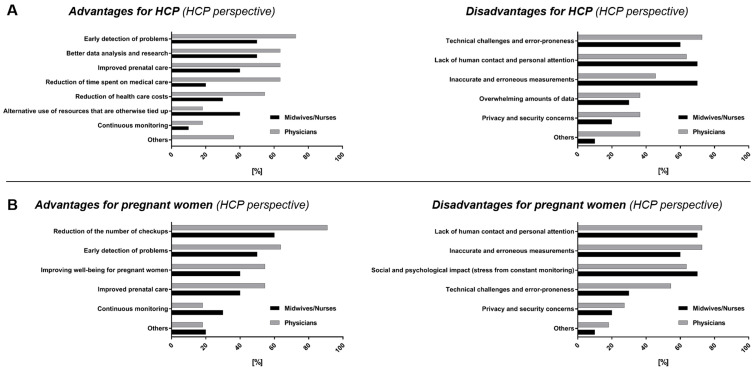
Perceived advantages and disadvantages of telemonitoring in pregnancy for (**A**) healthcare professionals in their daily work and (**B**) pregnant women, comparing the perspective of physicians (*n* = 11) and midwives/nurses (*n* = 10).

When questioned about the advantages and disadvantages of telemonitoring for pregnant women and themselves as healthcare professionals, responses from physicians and midwives/perinatal care nurses were compared, since these professionals oversee different aspects of care and may have differing views on remote monitoring. Accordingly, Figure 1 presents their opinions separated in two groups: Group 1 (physicians, *n* = 11) and Group 2 (midwives/nurses, *n* = 10).

For their everyday work, 72.7% of the 11 physicians surveyed recognized the potential of telemonitoring in the early detection of problems, with one physician emphasizing, “Reduction in perinatal morbidity and potentially even mortality.” (Figure 1A). However, only 50% of the 10 midwives/nurses perceive a benefit in this area. Sixty-three point six percent of the physicians, and 50% of the midwives/nurses see potential in telemonitoring for improving data analysis and supporting research. While 63,6% of the physicians surveyed see an advantage in the improved prenatal care and the reduction in time spent on medical care through telemonitoring, fewer than half of the midwives/nurses share this view (40% and 20%, respectively).

Fifty-four point five percent of the physicians surveyed see potential in reducing healthcare costs through telemonitoring, while only 30% of midwives/nurses do. However, 40% of midwives/nurses recognize the potential of telemonitoring to alternative use of resources that are otherwise tied up, compared to only 18.2% of physicians (Figure 1A).

From the healthcare professionals’ perspective, 90.9% of physicians and 60% of midwives/nurses see a benefit in the reduction in the number of checkups for pregnant women (Figure 1B). Furthermore, physicians as well as midwives/nurses recognize an advantage in early detection of problems (Physicians: 63.6%; midwives/nurses: 50%), improved prenatal care and well-being for pregnant women (Physicians: 54.5%; midwives/nurses: 40%) (Figure 1B). One respondent noted, “Resources are promoted, responsibility for oneself is increased.”

Physicians see disadvantages or risks of telemonitoring in their daily work primarily in the associated technical challenges and error-proneness (72.7%), followed by the lack of human contact and personal attention (63.6%) (Figure 1A). In their daily work, 70% of the midwives are particularly concerned about the risk of inaccurate and incorrect measurements and the lack of human contact and personal care through telemonitoring (Figure 1A). A midwife expressed concern about the additional effort required, noting that “telemedicine requires a different form of care and intensive training for both users and operators.”

Both professional groups identify key disadvantages of telemonitoring for pregnant women, including the lack of human contact and personal attention (Physicians: 72.7%; midwives/nurses: 70%), the risk of inaccurate or erroneous measurements (Physicians: 72.7%; midwives/nurses: 60%), and the potential social and psychological effects associated with continuous monitoring (Physicians: 63.6%; midwives/nurses: 70%) (Figure 1B). A midwife highlighted potential issues, stating: “Being alone with technology at home, feeling overwhelmed, and dealing with false alarms.”

When asked which type of wearable device would be most comfortable for women to use during pregnancy, all 20 respondents could envision a watch or bracelet, followed by clothing, such as T-shirts or underwear (70.0%) or a patch that can be stuck on the skin (50.0%). An abdominal belt was preferred by 45.0%, followed by an upper arm belt (30%). Only one respondent could imagine a headband.

**Figure 2:**
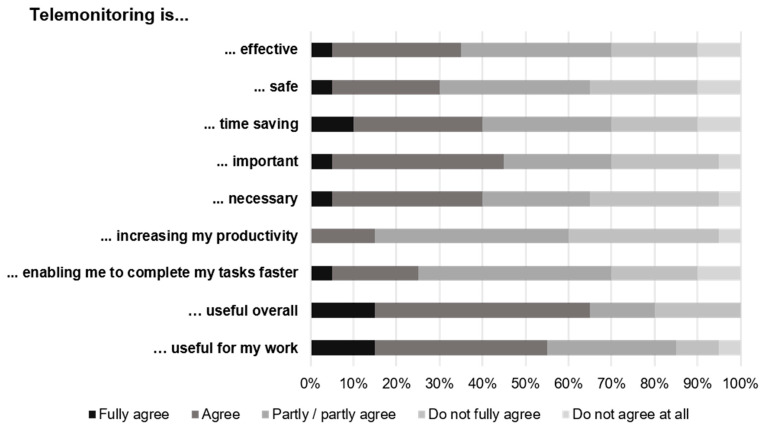
Personal attitudes of healthcare professionals (*n* = 20) toward telemonitoring.

Furthermore, 65.0% of the 20 respondents would consider telemonitoring useful overall and 55.0% useful for their work (fully agree/agree) (Figure 2). Forty percent of all respondents do not believe telemonitoring could increase their productivity and 35.0% believe telemonitoring is not necessary (Figure 2).

#### 3.1.2. Telemonitoring in Pregnant Women with and Without Risk Factors

Monitoring medical parameters in pregnant women enables the early identification of pregnancy-related complications. The assessment of the importance of medical parameters by physicians, nurses, and midwives separately for high-risk pregnancies (*n* = 21) and pregnancies without increased risk (*n* = 20) showed that significant differences exist only for the parameters “blood pressure” and “maternal heart rate” (Table 1).

Table 2 presents the respondents’ views on which clinical parameters should be accessible to the pregnant woman, her partner, primary care professionals, and hospital-based healthcare providers. Considering all clinical parameters listed in Table 2, the majority of healthcare professionals favor that the collected information should be available to the pregnant women themselves, both in high-risk and low-risk pregnancies (Table 2). More than half of the respondents indicated that for pregnant women with risk factors, this information should also be accessible to professionals in the hospital and professionals in primary healthcare (Table 2). For pregnant women without risk factors, nearly two-thirds of respondents recommended that the information be accessible to primary care professionals, while only one-third supported access for hospital-based professionals (Table 2).

##### Pregnant Women with Risk Factors

For pregnant women with risk factors, all healthcare professionals (*n* = 21) estimate that the parameters blood pressure, pain and contractions are relevant for pregnant women. Ninety-five point two percent of the respondents considered fetal movement and fetal growth to be important for pregnant women.

According to healthcare professionals, the parameters should be measured at different frequencies in pregnant women with risk factors. Most of the healthcare professionals indicated that weight (85.7%), urine sample (76.2%), fetal growth (71.4%), periodontitis (71.4%), physical activity (61.9%), respiration (61.9%), edema (57.1%), sleep (57.1%), and maternal heart rate (52.4%) should be measured at the same frequency as pregnancy checks. Among parameters to be measured between prenatal visits, blood pressure (66.7%), contractions (66.7%), fetal movements (66.7%), pain (47.6%), maternal heart rate variability (47.6%), and fetal heart rate (47.6%) were mentioned most often.

The frequency of measurements, which is reasonable for pregnant women with risk factors, is estimated by the healthcare professionals to be daily for the parameters of fetal movement (66.7%), blood pressure (52.4%), maternal heart rate (42.9%), respiration (38.1%), fetal heart rate (38.1%), physical activity (33.0%) and, respiration (38.1%), maternal heart rate (42.9%), variability of maternal heart rate (28.6%), fetal heart rate (38.1%), and fetal movement (66.7%). Weekly reasonable measurements should be taken for the parameters of weight (42.9%), sleep (33.3%), and fetal growth (33.3%), according to the healthcare professionals. In case of physical complaints of the mother, the parameters urine pain (57.1%), contractions (47.6%), edema (38.1%), sleep (33.3%) and urine (28.6%), edema (38.1%), pain (57.1%), contractions (47.6%), and sleep (33.3%) should be measured.

##### Pregnant Women Without Risk Factors

For pregnant women without risk factors, the healthcare professionals (*n* = 20) estimate that the parameters pain (90.0%), contractions (95.0%), pain (90.0%), and fetal movements (85.0%) are relevant from the pregnant woman’s point of view. According to healthcare professionals, all parameters should be measured at the same frequency as follow-up visits in pregnant women without risk factors.

In pregnant women without risk factors, most parameters (pain (75.0%), contractions (75.0%), blood pressure (25.0%), urine sample (25.0%), respiration (60.0%), periodontitis (60.0%), maternal heart rate variability (55.0%), edema (50.0%), pain (75.0%), contractions (75.0%), sleep (45.0%), maternal heart rate (45.0%), physical activity (40.0%), blood pressure (25.0%) and urine sample (25.0%), sleep (45.0%), respiration (60.0%), maternal heart rate (45.0%), maternal heart rate variability (55.0%), and periodontitis (60.0%)) should be measured only if there are symptoms. In addition, weight should be measured biweekly (30.0%), fetal heart rate (25.0%) weekly, and fetal movements (35.0%) daily.

In addition to the above factors, some healthcare professionals would also like to receive information on psychological well-being as well as depression (*n* = 4), blood glucose (*n* = 2), various blood values (*n* = 1), as well as nutrition (*n* = 1), cervix length (*n* = 1), and temperature (*n* = 1).

According to the healthcare professionals (multiple answers possible, *n* = 52), pregnant women with pregnancy complications (26.9%), as well as pregnant women with long distances to health services (26.9%) and pregnant women with risk factors (25.0%) would benefit most from telemonitoring applications. Seven point seven percent of responses indicate that all pregnant women could benefit, while 5.8% indicate that either women in preterm labor could benefit, or that there would be no benefit compared to existing systems. There was little concern that third parties could benefit from this (1.9%).

#### 3.1.3. Data Processing in the Process of Telemonitoring

Regarding the data processing in the process of telemonitoring, 75.0% of the interviewed healthcare professionals (*n* = 20) wish that a telemonitoring device would send the information directly to the healthcare professional, this should be performed during or between pregnancy checks if needed (95.0%).

While 25.0% of respondents agree that the pregnant woman’s partner(s) should also have access to the information collected by the remote monitoring device, 50.0% of respondents disagree. Being able to view the data in real time is something that 40.0% of respondents would like to see, while 35.0% are not sure about this. Sixty percent of the respondents would like the device to trigger an alarm if the collected readings are abnormal. In this case, the device should also provide the pregnant woman with information on what to do (85.0%).

**Figure 3:**
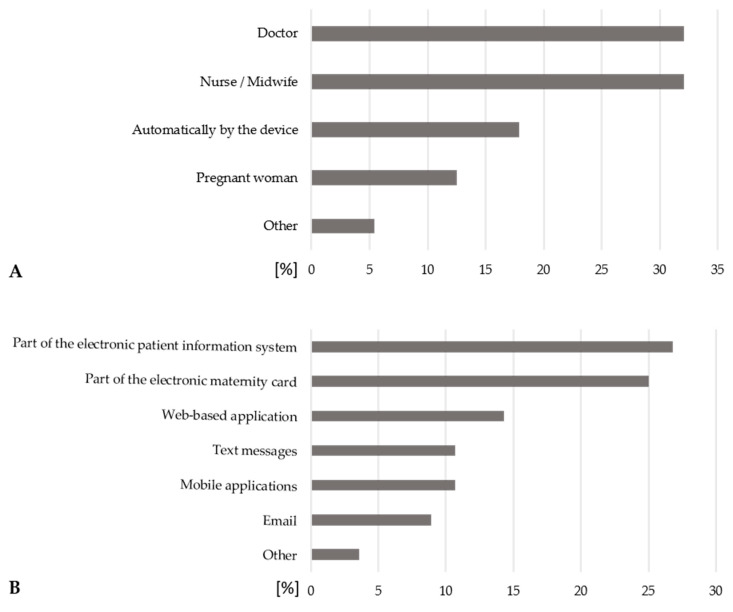
Preferences of healthcare professionals as to who should evaluate the data collected by the remote monitoring device (**A**) multiple answers possible, *n* = 56, and the format in which the healthcare professionals would like to receive the information (**B**) multiple answers possible, *n* = 56.

According to healthcare professionals, healthcare professionals should primarily evaluate the data (Figure 3A). One respondent suggested an “interdisciplinary team of physicians and midwives.” Furthermore, healthcare professionals believe that the information should be part of the electronic patient information system or the electronic maternity card (Figure 3B).

At the end of the questionnaire, respondents were invited to add any aspects of remote monitoring that they felt were missing from the survey. A midwife noted: “Monitoring always requires user training, so it takes more time initially and requires a lot of time for data review and evaluation. The bottom line is that there is probably no time savings, but in specific cases where indicated, seamless monitoring with better prediction is possible, making it modern and up-to-date, similar to continuous glucose monitoring (CGM). A physician criticized: “An ‘established’ method is assumed without these parameters ever having been investigated. The potential harm has been little questioned. I hope that well-designed, randomized studies will be conducted to help reduce current problems, such as subjective cardiotocography (CTG) evaluation.”

### 3.2. Oral Interview

To supplement the findings of the written survey, we further conducted oral interviews with the healthcare professionals. The following results summarize the transcribed findings of the oral interviews on remote monitoring during pregnancy and early labor.

#### 3.2.1. Aims of Prenatal Care and Monitoring

As the main aims of prenatal care and monitoring, the majority of respondents named medical care, advice as well as support (67.8%); multiple answers possible, total *n* = 59), followed by the aspect of education (10.4%), the health and well-being of mothers and children (8.5%), psychosocial support for women (6.8%), planning and risk management (5.1%) and the use of gentle medical procedures (1.7%).

#### 3.2.2. General Parameters During Pregnancy

The most important general parameters (*n* = 21 in total) during pregnancy according to the respondents were the subjective physical well-being (28.6%), followed by the subjective psychological well-being in general (14.3%), as well as in relation to an obstetrical event (14.3%).

#### 3.2.3. Medical Parameters and Information to Consider During Pregnancy

The three most important medical parameters and information to consider during pregnancy according to the respondents (multiple answers possible, *n* = 68 in total) for mothers were laboratory values (35.3%), followed by cardiovascular parameters (32.4%) and weight (11.8%).

For the fetuses (multiple answers possible, *n* = 41 in total), the parameters mentioned were growth and weight (43.9%), cardiac activity and heart rate (14.6%) as well as fetal movements, ultrasound examinations, malformations and measurement of the symphysis-fundus distance and abdominal circumference (7.3% each).

#### 3.2.4. Parameters Monitored Dependent on the Mothers’ Situation

The respondents were asked which parameters they would monitor in different situations in for women with pregnancy-related complications in the home environment (Table 3).

Blood pressure was considered to be the most important mother’s parameter to measure in women with pregnancy-induced hypertension without pre-eclampsia, intrauterine growth retardation, and status post intrauterine fetal death (Table 3). In the case of fetal malformation, the most important mother’s parameter to measure according to healthcare professionals was pain. In the event of a threatened preterm labor, healthcare professionals consider contractions to be the most important mother’s parameter to measure in the home environment (Table 3).

Concerning fetal parameters, healthcare professionals highlighted fetal growth, heart rate, and movement as the three key parameters to be monitored in the home environment in women with pregnancy-induced hypertension without pre-eclampsia, intrauterine growth restriction or status post intrauterine fetal death (Table 3). In the case of fetal malformation, the most important fetal parameters to measure according to healthcare professionals was growth, movement and malformation. Fetal parameters to measure in home environment in a threatened preterm labor, would be heart rate and movement (Table 3).

#### 3.2.5. Utilization of Home-Monitoring

If it were possible to monitor various parameters on an outpatient basis with the help of telemonitoring, 28.3% of responses indicated that there would be advantages or benefits of telemedicine (multiple answers possible, total *n* = 99). Twenty-seven point three percent of responses showed that personal contact and the specialist knowledge of medical staff were still important. Seventeen point two percent of responses expressed support for additional checks and monitoring.

More than half of the responses (57.9%) supported telemonitoring as a justifiable option (multiple responses allowed, total *n* = 38). Twenty-one point one percent indicated that remote monitoring could not be an option for monitoring pregnancy, while an equal proportion suggested that it could be an option to some extent.

Among the 27 responses (multiple answers possible), 59.3% agreed and provided justification that reducing in-person doctor visits during pregnancy was desirable. In contrast, 33.3% considered this to be only partially true, while 3.7% either disagreed or had no clear opinion on the matter.

#### 3.2.6. Desired Future Information

The parameters that respondents would like to have information about in the future included renal function values (*n* = 1), nutrition (*n* = 2), psychosocial parameters (*n* = 1), early detection of premature birth (*n* = 3), combination of blood pressure and weight (*n* = 1), vaginal pH (*n* = 2), continuous blood glucose monitoring (*n* = 1), knowledge about pregnancy and birth (*n* = 1), cervical measurement (*n* = 2), information on the pregnant woman’s professional environment (*n* = 1), information on the postpartum period (*n* = 1), metabolic support during pregnancy (*n* = 2), well-being of the woman (*n* = 1) and increased CTG monitoring at home (*n* = 1).

#### 3.2.7. Remote Monitoring During Labor

For fetal monitoring, the respondents named the parameters (multiple answers possible, *n* = 25 in total) heart rate (48.0%), fetal movements (24.0%) and CTG (16.0%) as most important to monitor during labor.

For the respondents, the three most important parameters for the mother during early labor (multiple answers possible, *n* = 50 in total) were the mother’s heart rate (24.0%), temperature (20.0%), and blood pressure (14.0%).

## 4. Discussion

### 4.1. Principal Findings

The findings of this study offer insights into the attitudes and acceptance of telemonitoring during pregnancy and early labor among healthcare professionals—including obstetricians, neonatologists, prenatal nurses, and midwives—working at the University Hospital Bern, Switzerland. The study also explored preferences for data processing in telemonitoring and the relevance of medical parameters in high-risk and low-risk pregnancies. To our knowledge, this is the first survey in Switzerland to explore healthcare professionals’ perspectives on telemonitoring in this context. This study employed an exploratory quantitative design with 21 respondents. Although the sample size was small, the findings may provide a foundation for larger-scale studies in the future.

Over half of the respondents expressed a positive attitude toward the use of telemonitoring during pregnancy and early labor. Nonetheless, a minority of participants reported opposition to its implementation.

The findings indicate notable differences in the perceived benefits of telemonitoring between physicians and midwives/nurses.

Furthermore, the study highlights differences in the perceived importance of medical parameters between high-risk and low-risk pregnancies and provides insights into healthcare professionals’ preferences for data management in telemonitoring.

### 4.2. Attitude and Acceptance of Telemonitoring

A majority of respondents expressed willingness to adopt telemonitoring in maternal care, highlighting its perceived benefits in improving care quality and accessibility. However, concerns about technical challenges, inaccurate or erroneous measurements, lack of human contact, and stress from constant monitoring are consistent with findings from other studies, emphasizing the need for careful implementation and appropriate safeguards to address these issues [21,24,28].

We found a notable difference in how physicians and midwives/nurses perceive the benefits of telemonitoring in clinical practice. While a majority of physicians recognize its potential for early detection of complications, reducing workload and prenatal visits, and lowering healthcare costs, midwives and nurses tend to be more reserved, with fewer acknowledging these advantages. Both groups, however, acknowledged its potential to aid early problem detection and enhance prenatal care and maternal well-being. In daily clinical practice, physicians primarily view technical challenges and the potential for errors as key disadvantages of telemonitoring. In contrast, midwives/nurses are particularly concerned about inaccurate measurements and the loss of personal contact and care, further highlighting the differing priorities and perceived risks between the two professional groups. Thus, our findings suggest that the professional background influences the perceived value of telemonitoring, with physicians generally more optimistic about its clinical and patient-centered benefits than midwives/nurses. This difference may be explained by variations in professional roles and responsibilities. Physicians often focus on diagnostic accuracy, efficiency, and clinical outcomes—areas where telemonitoring offers clear advantages, such as early detection of complications and improved data availability. In contrast, midwives and nurses place greater emphasis on direct patient interaction and the interpersonal aspects of care, which they may perceive as being reduced through digital tools. Consequently, their more cautious attitude likely reflects concerns about the potential loss of personal contact and the increased reliance on technology in maternal care. The issues displayed by midwives and nurses toward telemonitoring during pregnancy and early labor in this study aligns with findings reported in the existing literature [21,29,30]. Although, midwives generally saw potential in using videoconferencing during early labor and acknowledged the benefits, such as modernizing care, they raised concerns about privacy, for both themselves and the pregnant women, data accuracy and negative effects on the relationship between the mother-to-be and the professional [29,30]. Thus, efforts to encourage the use of telemedicine should especially involve nurses and midwives, as these groups appear more cautious toward its adoption, as also noted in the survey by Grassl et al., in Germany [21].

However our findings also show that, both physicians and midwives/nurses similarly acknowledge key drawbacks of telemonitoring for pregnant women. These include particularly the reduced human contact and personal attention, the risk of inaccurate measurements, and the possible social and psychological burdens linked to constant monitoring, potentially reflecting the importance that both physicians and midwives/nurses place on the human and relational aspects of maternity care. A recent study involving pregnant women with diabetes supports the concerns of healthcare professionals, showing that—even while recognizing the benefits of telemonitoring—these women experienced anxiety and insecurity when monitoring the fetal heart rate at home without direct support from healthcare staff [31]. While this study focused on healthcare professionals’ perspectives, it is essential to also consider the views and needs of pregnant women themselves. Our findings on professionals’ preferences and expectations have provided the basis for our ongoing research with pregnant women, aiming to directly explore their perspectives.

In addition to differences between professional groups, the literature also highlights a generational gap in attitudes toward telemedicine, with millennials—who frequently recognize the benefits of pregnancy monitoring apps—demonstrating greater enthusiasm for adopting these technologies [21]. However, the age of participants was not considered in this study, which may limit insights into potential generational differences in attitudes toward telemonitoring.

Overall, these findings highlight the importance of addressing concerns related to technology in maternal and neonatal care and improving access to high-quality devices for both patients and healthcare professionals—consistent with the results of studies conducted in other countries [24,32].

### 4.3. Telemonitoring in Pregnant Women with and Without Risk Factors

The study further highlights variations in the perceived importance of medical parameters in high-risk pregnancies compared to pregnancies without increased risk. Notably, blood pressure and maternal heart rate were considered significantly more important in high-risk pregnancies, likely due to their strong association with complications that can result in adverse outcomes for both mother and child [33]. Additionally, healthcare professionals identified blood pressure, uterine contractions, and fetal movements as key clinical parameters that should be monitored between regular prenatal visits in high-risk pregnancies. By contrast, for low-risk pregnant women, most parameters should be monitored only if symptoms arise. The benefits of remote monitoring for high-risk pregnancies have been already recognized in prior research by both midwives and obstetricians [34].

Healthcare professionals suggest that certain parameters should be measured at different frequencies, depending on the presence of risk factors. The choice of measurement frequency reflects the healthcare professionals’ consideration of the unique needs and potential risks associated with high-risk pregnancies.

These results reinforce the view that remote monitoring strategies should be tailored to risk profiles, supporting earlier detection of complications and more personalized prenatal care, as recognized by both midwives and obstetricians in prior studies [9,24,35,36].

### 4.4. Data Processing in Telemonitoring

In this study, healthcare professionals expressed a strong preference for telemonitoring data to be sent directly to them, either during or between prenatal visits. Respondents emphasized that data should primarily be evaluated by healthcare professionals.

While healthcare professionals generally advocate that collected data should be visible to pregnant women, there is a notable reluctance to provide access to the pregnant woman’s partner(s). These concerns mirror those previously reported in the literature regarding privacy and data protection [29] highlighting the need for robust data protection measures. These reservations may have resulted from the lack of comprehensive privacy policies in current mobile pregnancy monitoring applications [37].

Healthcare professionals also express interest in real-time data visualization but are divided on the desirability of this feature, suggesting a need for a balance between real-time access and privacy.

Furthermore, healthcare professionals agree that the telemonitoring device should trigger an alarm for abnormal readings and provide guidance to the pregnant woman on appropriate actions. This proactive approach would enhance patient safety and empower pregnant women to respond effectively to critical situations, while also addressing previously documented concerns regarding usability, anxiety, and data reliability [38]. However, it should be noted that there are currently no guidelines on how to handle which data in which situations. These need to be developed before these systems are used. On the other hand, real-time feedback from the system is a factor that increases its popularity.

### 4.5. Strengh of the Study

A key strength of this study is its exploratory design, which allowed for a comprehensive understanding of healthcare professionals’ attitudes and acceptance of telemonitoring. By including multiple professional groups—physicians, midwives, and prenatal care nurses—the study captures diverse perspectives from those directly involved in maternal and fetal care. The use of both questionnaires and in-depth interviews enabled the collection of nuanced data, providing insights into not only general trends but also the reasoning and context behind participants’ views. Furthermore, as one of the first studies to examine telemonitoring attitudes in Switzerland, these findings can serve as a foundation for future, larger-scale investigations.

### 4.6. Limitations of the Study

Due to the interview-based design and intra-institutional recruitment, there is a possibility of response bias in the answers. While the written surveys were anonymous, the interviews were not, so a bias stemming from, for example, socially desirable responses cannot be ruled out. To minimize this, participation was voluntary, and the value of honest, unbiased feedback was emphasized. Additionally, all participants were aware of the study’s objective, and blinding was not possible. During the transcription process, the merging of individual pieces of information may have led to the loss of some details.

It should be noted that the total number of participants in this study is comparable to other studies [39,40,41]. To the best of our knowledge, only two studies included a higher number of respondents, yet [21,41]. Although smaller sample sizes are common in qualitative research, as no new important information is expected to come up in additional interviews, our findings should be regarded as exploratory and hypothesis-generating rather than definitive. Our findings warrant validation through additional studies using a larger sample.

## 5. Conclusions

This study provides insights into the healthcare professionals’ attitudes and acceptance regarding telemonitoring during pregnancy and early labor at the University Hospital Bern, Switzerland. It highlights differences between physicians and midwives/nurses and between high-risk and low-risk pregnancies. While telemonitoring is recognized as a promising tool for early detection of complications and personalized prenatal care, concerns regarding data accuracy, reduced personal contact and usability remain. Future studies should involve larger and more diverse samples, including pregnant women to validate these findings. Additionally, clear clinical guidelines for data use, alarm management, and privacy protection should be developed. Training programs should be strengthened to enhance confidence and usability among healthcare professionals.

## Figures and Tables

**Table 1:** Assessment of the importance of medical parameters in high- and low-risk pregnancies.

Parameter	High-Risk Pregnancy	Very Important		Important		Partly/Partly		Rather Unimportant		Unimportant		***p***
		*n*	%	*n*	%	*N*	%	*n*	%	*N*	**%**	
Blood pressure	Yes	14	**66.7**	6	28.6	1	4.8	0	0	0	0	**0.007**
	No	5	25.0	11	**55.0**	2	10.0	2	10.0	0	0	
Weight	Yes	4	19.1	9	**42.9**	5	23.8	3	14.3	0	0	0.498
	No	2	10.0	9	**45.0**	5	25.0	3	15	1	5	
Urine sample	Yes	6	28.6	5	23.8	8	**38.1**	2	9.5	0	0	0.132
	No	3	15.0	5	25.0	7	**35.0**	4	20.0	1	5.0	
Edema	Yes	6	**28.6**	5	23.8	6	**28.6**	4	19.1	0	0	0.075
	No	2	10.0	5	25.0	7	**35.0**	5	25.0	1	5.0	
Pain	Yes	7	33.3	10	**47.6**	4	19.1	0	0	0	0	0.209
	No	5	25.0	9	**45.0**	3	15.0	2	10.0	1	5.0	
Contractions	Yes	9	**42.9**	9	**42.9**	3	14.3	0	0	0	0	0.428
	No	8	**40.0**	8	**40.0**	2	10.0	1	5.0	1	5.0	
Physical activity	Yes	2	9.5	8	**38.1**	8	**38.1**	3	14.3	0	0	0.356
	No	3	15.0	7	**35.0**	3	15.0	6	30.0	1	5.0	
Sleep	Yes	1	4.8	11	**52.4**	6	28.6	3	14.3	0	0	0.609
	No	4	20.0	6	**30.0**	4	20.0	5	25.0	1	5.0	
Respiration	Yes	1	4.8	6	28.6	10	**47.6**	3	14.3	1	4.8	0.713
	No	2	10.0	6	30.0	2	10.0	8	**40.0**	2	10.0	
Maternal heart rate	Yes	3	14.3	8	**38.1**	7	33.3	3	14.3	0	0	**0.023**
	No	2	10.0	5	25.0	5	25.0	7	**35.0**	1	5.0	
Variability of maternal heart rate (sign of stress)	Yes	2	9.5	9	**42.9**	7	33.3	3	14.3	0	0	0.150
	No	1	5.0	6	30.0	7	**35.0**	5	25.0	1	5.0	
Fetal heart rate	Yes	8	38.1	10	**47.6**	3	14.3	0	0	0	0	0.399
	No	6	30.0	7	**35.0**	3	15.0	4	20.0	0	0	
Fetal movement	Yes	8	38.1	10	**47.6**	2	9.5	1	4.8	0	0	0.751
	No	10	**50.0**	7	35.0	1	5.0	2	10.0	0	0	
Fetal growth	Yes	9	**42.9**	8	38.1	4	19.1	0	0	0	0	0.128
	No	6	30.0	10	**50.0**	1	5.0	2	10.0	1	5.0	
Periodontitis	Yes	0	0	9	**42.9**	3	14.3	7	33.3	2	9.5	0.475
	No	3	15.0	4	20.0	5	**25.0**	4	20.0	4	20.0	

Pregnancies with high-risk factors (*n* = 21) versus pregnancies without increased risk (*n* = 20); Highest values within each parameter are shown in bold; Bold values in column *p* indicate statistical significance level of *p* < 0.05 (ANOVA).

**Table 2:** Respondents’ views on clinical parameter access.

Parameter	High-Risk Pregnancy	Pregnant Woman		Partner		Primary Health Care		Hospital		**Others**	
		*n*	%	*n*	%	*n*	%	*n*	**%**	***n***	**%**
Blood pressure	Yes	17	81.0	4	19.1	16	76.2	14	66.7	1	4.8
	No	16	80.0	1	5.0	15	75.0	8	40.0	1	5.0
Weight	Yes	17	81.0	0	0.0	13	61.9	13	61.9	0	0.0
	No	17	85.0	0	0.0	13	65.0	8	40.0	0	0.0
Urine sample	Yes	15	71.4	0	0.0	12	57.1	15	71.4	1	4.8
	No	13	65.0	0	0.0	14	70.0	8	40.0	0	0.0
Edema	Yes	17	81.0	0	0.0	10	47.6	13	61.9	2	9.5
	No	14	70.0	0	0.0	12	60.0	7	35.0	1	5.0
Pain	Yes	17	81.0	3	14.3	11	52.4	16	76.2	2	9.5
	No	17	85.0	3	15.0	15	75.0	6	30.0	1	5.0
Contractions	Yes	17	81.0	4	19.1	11	52.4	17	81.0	1	4.8
	No	16	80.0	3	15.0	11	55.0	9	45.0	1	5.0
Physical activity	Yes	20	95.2	1	4.8	8	38.1	10	47.6	2	9.5
	No	18	90.0	0	0.0	11	55.0	5	25.0	1	5.0
Sleep	Yes	19	90.5	2	9.5	11	52.4	9	42.9	1	4.8
	No	18	90.0	1	5.0	12	60.0	4	20.0	1	5.0
Respiration	Yes	17	81.0	0	0.0	11	52.4	9	42.9	0	0.0
	No	18	90.0	0	0.0	10	50.0	5	25.0	0	0.0
Maternal heart rate	Yes	15	71.4	1	4.8	12	57.1	10	47.6	0	0.0
	No	15	75.0	0	0.0	13	65.0	7	35.0	0	0.0
Variability of maternal heart rate (sign of stress)	Yes	12	57.1	0	0.0	13	61.9	10	47.6	0	0.0
	No	14	70.0	0	0.0	12	60.0	6	30.0	1	5.0
Fetal heart rate	Yes	15	71.4	1	4.8	11	52.4	17	81.0	1	4.8
	No	15	75.0	0	0.0	12	60.0	9	45.0	1	5.0
Fetal movement	Yes	19	90.5	3	14.3	10	47.6	17	81.0	1	4.8
	No	19	95.0	3	15.0	11	55.0	9	45.0	1	5.0
Fetal growth	Yes	15	71.4	2	9.5	12	57.1	16	76.2	1	4.8
	No	16	80.0	2	10.0	13	65.0	8	40.0	1	5.0
Periodontitis	Yes	16	76.2	0	0.0	15	71.4	8	38.1	2	9.5
	No	15	75.0	0	0.0	13	65.0	3	15.0	2	10.0
Over all parameters	Yes	248	78.7	21	6.6	176	55.9	194	61.6	15	4.8
	No	241	80.3	13	4.3	187	62.3	102	34.0	12	4.0

Pregnancies with high-risk factors (*n* = 21) versus pregnancies without increased risk (*n* = 20).

**Table 3:** Frequency of key home monitoring parameters (maternal vs. fetal) by clinical situation.

**A. Mothers’ parameters**					
Parameter (x/%)	Situation				
	PIHw/oPE (*n* = 99)	IUGR (*n* = 70)	FM (*n* = 23)	s/pIUFD (*n* = 37)	PTL (*n* = 53)
Blood pressure	23/23.2	18/25.7	5/21.7	11/29.7	7/13.2
Heart rate	6/6.1	4/5.7	2/8.7	2/5.4	4/7.5
Edema	14/14.1	9/12.9	1/4.3	1/2.7	2/3.8
Proteinuria/urine	14/14.1	9/12.9	2/8.7	2/5.4	3/5.7
Physical activity	6/6.1	4/5.7	2/8.7	0/0	2/3.8
Sleep	8/8.1	4/5.7	1/4.3	2/5.4	4/7.5
Weight	13/13.1	8/11.4	1/4.3	3/8.1	3/5.7
Pain	8/8.1	6/8.6	7/30.4	9/24.3	9/17.0
Contractions	4/4.0	4/5.7	2/8.7	5/13.5	16/30.2
Breathing rate	2/2.0	2/2.9	0/0	0/0	2/3.8
Blood sugar	0/0	2/2.9	0/0	1/2.7	0/0
Customized parameters	1/1.0	0/0	1/4.3	1/2.7	1/1.9
**B. Fetal parameters**					
Parameter (x/%)	Situation				
	PIHw/oPE (*n* = 26)	IUGR (*n* = 42)	FM (*n* = 37)	s/pIUFD (*n* = 37)	PTL (*n* = 21)
Growth	8/30.8	14/33.3	8/21.6	5/13.5	4/19.0
Heart rate	7/26.9	12/28.6	6/16.2	10/27.0	6/28.6
Movement	9/34.6	12/28.6	10/27.0	12/32.4	6/28.6
CTG	1/3.8	1/2.4	0/0	1/2.7	0/0
Ultrasound	1/3.8	2/4.8	1/2.7	0/0	0/0
Malformation	0/0	0/0	8/21.6	1/2.7	0/0
Fetal heart sounds	0/0	1/2.4	1/2.7	1/2.7	0/0
Fetal monitoring in general	0/0	0/0	3/8.1	4/10.8	4/19.0
Depending on cause and time of s/pIUFD	0/0	0/0	0/0	3/8.1	0/0
Dopton	0/0	0/0	0/0	1/2.7	1/4.8

PIHw/oPE–pregnancy-induced hypertension without pre-eclampsia; IUGR–intrauterine growth retardation; FM–fetal malformation; s/pIUFD–status post intrauterine fetal death; PTL–threatened preterm labor; Frequency is shown as (x/%)—where x is how often a parameter was mentioned, compared to the total number of responses (n) (multiple answers possible).

## Data Availability

The datasets used and/or analyzed during the current study are available from the corresponding author on reasonable request.

## Supplementary Materials

The following supporting information can be downloaded at: https://www.mdpi.com/article/10.3390/ijerph22111753/s1, Supplementary File S1: Questionnaire—Healthcare Professionals’ Perceptions and Acceptance of Telemonitoring During Pregnancy and Early Labour: A Single-Centre Survey, Supplementary File S2: Interview—Healthcare Professionals’ Perceptions and Acceptance of Telemonitoring During Pregnancy and Early Labour: A Single-Centre Survey.
