# Supplementary material for: Healthcare Professionals’ Perceptions and Acceptance of Telemonitoring During Pregnancy and Early Labor: A Single-Center Survey
Source: Int J Environ Res Public Health. 2025 Nov 19;22(11):1753. doi: 10.3390/ijerph22111753 (PMC12652219; doi:10.3390/ijerph22111753)
Supplement: Supplementary file 1 [file ijerph-22-01753-s001.zip › ijerph-3892774-supplementary.pdf]

Supplementary File S1

Questionnaire - Healthcare Professionals' Perceptions and Acceptance of Telemonitoring During Pregnancy and Early Labor: A Single-Center Survey

A Background Information on the Specialist

1. How old are you? ______ years

2. What is your highest level of education?
   - Technical secondary school certificate
   - Technical maturity High school diploma
   - Federal vocational baccalaureate (EFZ)
   - Vocational maturity Federal vocational certificate (EBA)
   - Diploma
   - Higher Technical School (HF)
   - Professional examination (BP)
   - Higher professional examination (HFP)
   - Bachelor's / Master's degree from a University of Applied Sciences / Pedagogical University Bachelor's / Master's degree from a University Doctorate / PhD from a University
   - Other (please specify) ____

3. What is your profession?

4. Where is your current workplace located?
   - University hospital / Hospital
   - Private practice

5. How many years of professional experience do you have at your current workplace?
   ______ years

6. How many years of overall experience do you have in the healthcare sector?
   ______ years

B Options, Preferences, and Requirements related to Remote Monitoring

7. Do you endorse remote monitoring during pregnancy?
   - No
   - Yes
   - I don't know / No opinion

8. Do you endorse remote monitoring in the early stages of labor (onset of contractions, premature rupture of membranes, latent phase, possibly outpatient labor induction)?
   - No
   - Yes
   - I don't know / No opinion

9. Do you think pregnant women should use remote monitoring?
    - No
    - Yes
    - I don't know / No opinion

10. Can you explain why not? ____

11. What benefits or potentials do you see in remote monitoring of pregnant women? (Multiple answers possible)

<u>A) for your daily work</u>

- Continuous monitoring
- Early detection of problems
- Improved prenatal care
- Reduction of time spent on medical care
- Reduction of healthcare costs
- Other use of otherwise tied resources (personnel, time, etc.)
- Better data analysis and research
- Other (please specify): ________________________________

<u>B) for pregnant women</u>

- Continuous monitoring
- Early detection of problems
- Improved prenatal care
- Reduced doctor visits
- Enhancement of well-being for pregnant women
- Other (please specify): ________________________________

12. What disadvantages or risks do you see in remote monitoring of pregnant women? (Multiple answers possible)

<u>A) for your daily work</u>

- Inaccurate or faulty measurements
- Lack of human contact and personal care
- Privacy and security concerns
- Technical challenges and error susceptibility
- Overwhelming amount of data
- Other (please specify): ________________________________

<u>B) for pregnant women</u>

- Inaccurate or faulty measurements
- Lack of human contact and personal care
- Privacy and security concerns
- Technical challenges and error susceptibility
- Social and psychological impacts (stress from constant monitoring, fears, etc.)
- Other (please specify): ________________________________

13. In your opinion, are there risks if pregnant women do not use the remote monitoring device correctly
(e.g., incorrect data, impact on maternal or fetal well-being)?
   - o No
   - o Yes
   - o I don't know / No opinion

C Assessment of Parameters in Pregnant Women <u>with</u> Risk Factors

Imagine that a pregnant woman with risk factors is to be monitored during pregnancy.
Please evaluate each parameter based on the given criteria regarding a high-risk pregnancy.
Check what you believe applies or write your response in the open box.

14. How important do you find the following parameters for monitoring pregnant women with risk factors?

	Very important	Important	Partly / partly	Rather unimportant	Unimportant
Blood pressure					
Weight					
Urine sample					
Edema					
Pain					
Contractions					
Physical activity					
Sleep					
Respiration					
Maternal heart rate					
Variability of maternal heart rate (sign of stress)					
Fetal heart rate					
Fetal movement					
Fetal growth					
Periodontitis					

15. Who should have information about pregnant women with risk factors? (Multiple answers possible)

	Pregnant woman	Partner	Healthcare professionals in primary medical care	Healthcare professionals in the hospital	Other
Blood pressure					
Weight					
Urine sample					
Edema					

Pain					
Contractions					
Physical activity					
Sleep					
Respiration					
Maternal heart rate					
Variability of maternal heart rate (sign of stress)					
Fetal heart rate					
Fetal movement					
Fetal growth					
Periodontitis					
Other (please specify parameter and associated response)					

16. How relevant do you consider the information on each parameter for pregnant women with risk factors from the perspective of the pregnant woman?

	Very relevant	Relevant	Partly / partly	Not so relevant	Not relevant at all
Blood pressure					
Weight					
Urine sample					
Edema					
Pain					
Contractions					
Physical activity					
Sleep					
Respiration					
Maternal heart rate					
Variability of maternal heart rate (sign of stress)					
Fetal heart rate					
Fetal movement					
Fetal growth					
Periodontitis					

17. In which timeframe should each parameter be measured at home for pregnant women with risk factors? (Multiple answers possible)

	1st trimester	2nd trimester	3rd trimester
Blood pressure			

Weight			
Urine sample			
Edema			
Pain			
Contractions			
Physical activity			
Sleep			
Respiration			
Maternal heart rate			
Variability of maternal heart rate (sign of stress)			
Fetal heart rate			
Fetal movement			
Fetal growth			
Periodontitis			
Other (please specify parameter and associated response)			

18. How often should the parameter be measured for pregnant women with risk factors?

	With the same frequency as regular check-ups	Also between regular check-ups	Other (please specify)
Blood pressure			
Weight			
Urine sample			
Edema			
Pain			
Contractions			
Physical activity			
Sleep			
Respiration			
Maternal heart rate			
Variability of maternal heart rate (sign of stress)			
Fetal heart rate			
Fetal movement			
Fetal growth			
Periodontitis			
Other (please specify parameter and associated response)			

19. What is acceptable for pregnant women with risk factors regarding the frequency of measurements?

Blood pressure ____________________
Weight ____________________
Urine sample ____________________
Edema ____________________
Pain ____________________
Contractions ____________________
Physical activity ____________________
Sleep ____________________
Respiration ____________________
Maternal heart rate ____________________
Variability of maternal heart rate (sign of stress) ____________________
Fetal heart rate ____________________
Fetal movement ____________________
Fetal growth ____________________
Periodontitis ____________________

D Assessment of Parameters in Pregnant Women without Risk Factors

Imagine that a pregnant woman without risk factors is to be monitored during pregnancy.
Please evaluate each parameter based on the given criteria regarding a high-risk pregnancy.
Check what you believe applies or write your response in the open box.

20. How important do you find the following parameters for monitoring pregnant women without risk factors?

	Very important	Important	Partly / partly	Rather unimportant	Unimportant
Blood pressure					
Weight					
Urine sample					
Edema					
Pain					
Contractions					
Physical activity					
Sleep					
Respiration					
Maternal heart rate					
Variability of maternal heart rate (sign of stress)					
Fetal heart rate					
Fetal movement					
Fetal growth					
Periodontitis					

21. Who should have information about pregnant women without risk factors? (Multiple answers possible)

	Pregnant woman	Partner	Healthcare professionals in primary medical care	Healthcare professionals in the hospital	Other
Blood pressure					
Weight					
Urine sample					
Edema					
Pain					
Contractions					
Physical activity					
Sleep					
Respiration					
Maternal heart rate					
Variability of maternal heart rate (sign of stress)					
Fetal heart rate					

Fetal movement					
Fetal growth					
Periodontitis					
Other (please specify parameter and associated response)					

22. How relevant do you consider the information on each parameter for pregnant women without risk factors from the perspective of the pregnant woman?

	Very relevant	Relevant	Partly / partly	Not so relevant	Not relevant at all
Blood pressure					
Weight					
Urine sample					
Edema					
Pain					
Contractions					
Physical activity					
Sleep					
Respiration					
Maternal heart rate					
Variability of maternal heart rate (sign of stress)					
Fetal heart rate					
Fetal movement					
Fetal growth					
Periodontitis					

23. In which timeframe should each parameter be measured at home for pregnant women without risk factors? (Multiple answers possible)

	1st trimester	2nd trimester	3rd trimester
Blood pressure			
Weight			
Urine sample			
Edema			
Pain			
Contractions			
Physical activity			
Sleep			
Respiration			

Maternal heart rate			
Variability of maternal heart rate (sign of stress)			
Fetal heart rate			
Fetal movement			
Fetal growth			
Periodontitis			
Other (please specify parameter and associated response)			

24. How often should the parameter be measured for pregnant women without risk factors?

	With the same frequency as regular check-ups	Also between regular check-ups	Other (please specify)
Blood pressure			
Weight			
Urine sample			
Edema			
Pain			
Contractions			
Physical activity			
Sleep			
Respiration			
Maternal heart rate			
Variability of maternal heart rate (sign of stress)			
Fetal heart rate			
Fetal movement			
Fetal growth			
Periodontitis			
Other (please specify parameter and associated response)			

25. What is acceptable for pregnant women without risk factors regarding the frequency of measurements?

Blood pressure ______________________
Weight ______________________
Urine sample ______________________
Edema ______________________
Pain ______________________

Contractions ____________________
Physical activity ____________________
Sleep ____________________
Respiration ____________________
Maternal heart rate ____________________
Variability of maternal heart rate (sign of stress) ____________________
Fetal heart rate ____________________
Fetal movement ____________________
Fetal growth ____________________
Periodontitis ____________________

26. Are there other parameters that you believe should be measured during pregnancy?
____

E Data Management

27. How should the information collected with the remote monitoring device be managed?

28. The device should send the information directly to healthcare professionals.
    - No
    - Yes
    - I don't know

29. The device should transmit information to healthcare professionals for the pregnant woman as needed during or between prenatal check-ups.
    - No
    - Yes
    - I don't know

30. The partner of the pregnant woman should also have access to the information collected with the remote monitoring device.
    - No
    - Yes
    - I don't know

F Data Processing

31. It would be important to view the data in real-time.
    - No
    - Yes
    - I don't know

32. The device should trigger an alarm if the collected measurements are abnormal.
    - No
    - Yes
    - I don't know

33. In this case, the device should also provide information or guidance to the pregnant woman on what to do.
    - No
    - Yes
    - I don't know

34. Who should evaluate the data collected with the remote monitoring device? (Multiple answers possible)
    - Nurse/midwife
    - Doctor
    - Pregnant woman
    - Automatically by the device

- Other (please specify): ____

G Requirements for the Remote Monitoring Device

35. In what format would you like to receive the information? (Multiple answers possible)
    - o Mobile application
    - o Web-based application
    - o Text messages
    - o Email
    - o Part of the electronic maternity card
    - o Part of the electronic patient information system
    - o Other (please specify): ____________________

36. What type of wearable device do you think would be most comfortable for pregnant women to use during pregnancy? (Multiple answers possible)
    - o Clothing (T-shirt or similar)
    - o Belt
    - o Underwear, such as a sports bra
    - o Belly belt
    - o Watch/bracelet
    - o Upper arm strap
    - o Patch applied directly to the skin
    - o Headband
    - o Other (please specify): ____________________

H General Questions about Remote Monitoring Devices

37. Have you ever used a remote monitoring device? (e.g., for monitoring pregnant women or fetal well-being)
    - o No
    - o Yes

38. What device or application have you used? ____________________

39. Did you find the application or device useful?
    - o No
    - o Yes

40. Why did you find it useful? ____________________

41. Why did you not find it useful?

    ____

42. Who, in your opinion, would benefit the most from remote monitoring during pregnancy? (Multiple answers possible)
    - o All pregnant women
    - o Pregnant women with risk factors

- Pregnant women with pregnancy complications
- Pregnant women with a significant distance to healthcare services
- Pregnant women with premature contractions
- I see no additional benefit of remote monitoring compared to existing systems.
- Other (please specify): ____

I Attitude towards Remote Monitoring

43. The use of the remote monitoring system…

	Completely agree	Agree	Slightly / slightly	Neither agree nor disagree	Not entirely agree	Not at all agree
… would be useful for my work						
… would be useful overall						
… would enable me to complete my tasks faster						
… would increase my productivity						
… is necessary						
… is important						
… is time-saving						
… is secure						
… is effective						

44. What concerns do you have regarding the use of a remote monitoring system or similar device? (Multiple answers possible)
    - o Privacy
    - o Discomfort/pain for the pregnant woman
    - o Discomfort/pain for the fetus
    - o Overwhelming amount of data for the pregnant woman
    - o Overwhelming amount of data for healthcare professionals
    - o Incorrect use of the device
    - o Increased stress
    - o Additional time spent on evaluating monitored data
    - o Issues related to the functionality of the technology
    - o Other (please specify): ____

45. What hopes do you associate with the use of a remote monitoring system or a similar device? (Multiple answers possible)
    - o I will be able to monitor the pregnant woman and the fetus more accurately
    - o I will receive more information to support my decision-making and counseling
    - o Remote monitoring could alleviate the concerns of a pregnant woman
    - o The remote monitoring system could possibly reduce the number of prenatal check-ups
    - o The remote monitoring system could possibly reduce the need for hospitalization or shorten hospital stays
    - o Other (please specify): ____

46. Is there anything you missed in the interview that you would like to mention, or would you like to further comment on the NewLife project for remote monitoring?

Supplementary File S2

Interview - Healthcare Professionals' Perceptions and Acceptance of Telemonitoring During Pregnancy and Early Labor: A Single-Center Survey

Understanding current practices and workflows during pregnancy and early labor (20 minutes)

A Pregnancy

47. What do you consider to be the main aims of prenatal care/monitoring?

48. In your view, which key parameters or aspects should be considered when monitoring a pregnancy (e.g., frequency of check-ups, equipment used, professionals involved)?

49. Imagine the following situations:
    - a) a pregnant woman with pregnancy-induced hypertension (without preeclampsia), or
    - b) a pregnant woman with intrauterine growth restriction (IUGR), or
    - c) a pregnant woman with a fetal malformation of any kind, or
    - d) a pregnant woman with status post intrauterine fetal death (IUFD), or
    - e) a pregnant woman presenting with threatened preterm labor.

    Question: What and how frequently would you monitor this patient at home (e.g., fetal heart rate, fetal movements, maternal blood pressure, body temperature, etc.)? If possible, please provide a statement for each of the scenarios.

    Which other parameters, not mentioned in the questionnaire or here, do you consider relevant or important?

50. How do you determine whether a sign of a suspected complication during pregnancy requires particular attention or intensified monitoring? And how do you follow up in such a case?

51. Imagine you could monitor various parameters in an outpatient setting: How would the collected information (e.g., daily blood pressure profiles, fetal movement tracking) influence the management or care pathway of the pregnant woman?
    - a) Would you consider the remote monitoring system as a replacement for, or an addition to, the standard antenatal visits in high-risk pregnancies?
    - b) Do you have any ideas on how pregnancy monitoring could be planned, specifically a detailed workflow or flowchart for combined monitoring?

B Early Labor

1. From your perspective, is remote monitoring appropriate for patients with a history of spontaneous rupture of membranes without contractions, or during the early phase of labor induction, irrespective of the induction method?

2. What questions arise regarding the monitoring of the early phase of labor (latent phase, early active phase, premature spontaneous rupture of membranes without contractions)?
    a) Which parameters would you monitor, and at what intervals?

3. Is there anything related to labor monitoring that has not yet been mentioned?

Understanding opportunities for improvement (20 minutes)

A Pregnancy

1. What improvements could generally be made in prenatal care and monitoring of the fetus and mother for both low-risk and high-risk pregnancies?
    a) Would remote monitoring be an option?
    b) Are there any information or parameters related to pregnancy monitoring that you would like to have but are currently not available?

2. What type of remote monitoring could potentially reduce the number of doctor visits?
    a) From your perspective, would this even be desirable?
    b) If yes, in which situation?

B Early Labor

1. Imagine the following situations:
    a) a pregnant woman with spontaneous rupture of membranes without contractions
    b) a pregnant woman undergoing labor induction
    c) a pregnant woman in the early active phase of labor

   Question: What type of monitoring would allow her to remain safely at home for as long as possible?

Understanding user requirements and expectations for remote monitoring (15 minutes)

1. What opportunities and challenges do you see if pregnancy care is shifted more from the hospital or care unit to the home setting?

2. Have you ever used a remote monitoring device as part of pregnancy monitoring?
    a) What were/are the benefits?
    b) What were/are the challenges or problems?

3. What are the key requirements for remote monitoring in pregnancy care (e.g., technical features, visualization, instructions, user-friendliness)?
   What would be the ideal device/system from your point of view?

4. What type of wearable device do you think would be most comfortable for pregnant women to wear or use during pregnancy and/or in the early phase of labor (e.g., clothing, belt, underwear, abdominal band, skin sensor/patch, watch, etc.)?
